# Impairment of motor coordination and interneuron migration in perinatal exposure to glufosinate-ammonium

**DOI:** 10.1038/s41598-020-76869-7

**Published:** 2020-11-26

**Authors:** Kyung-Tai Kim, Ye-Jung Kwak, Su-Cheol Han, Jeong Ho Hwang

**Affiliations:** Jeonbuk Branch Institute, Korea Institute of Toxicology, 30 Baekhak1-gil, Jeongeup, Jeollabuk-do 56212 Republic of Korea

**Keywords:** Developmental biology, Developmental neurogenesis

## Abstract

Glufosinate-ammonium (GLA) is a broad-spectrum herbicide for agricultural weed control and crop desiccation. Due to many GLA-resistant crops being developed to effectively control weeds and increase harvest yields, herbicide usage and the residual GLA in food has increased significantly. Though perinatal exposure by the residual GLA in food might affect brain development, the developmental neurotoxicity of GLA is still unclear. Therefore, this study aimed to investigate the effects of perinatal exposure to GLA on cortical development. The analysis revealed that perinatal GLA exposure altered behavioral changes in offspring, especially motor functional behavior. Moreover, perinatal GLA exposure affected cortical development, particularly by disrupting interneuron migration. These results provide new evidence that early life exposure to GLA alters cortical development.

## Introduction

During brain development, cortical neurogenesis is tightly controlled and includes neural progenitor differentiation, neuronal migration, and neural circuit formation via axon and dendrite extension^1^. Cortical layers consist of excitatory cortical projection neurons and inhibitory interneurons; their developmental processes are completely different. Excitatory cortical projection neurons originate from the cortical mantle zone and radially migrate to the cortex, but inhibitory interneurons originate from the ganglionic eminence and tangentially migrate to the cortex^2^. Since each process of cortical development has a vital role for proper brain function, mistakes in these events cause brain function difficulties. Especially, about 25% of interneurons are cortical neurons and modulate brain activity; an abnormal inhibitory interneuron circuit formation can cause multiple neurodevelopmental disorders in humans^3–6^.

It is well known that environmental hazard factors influence cortical development and increase neurodevelopmental disorders. Neurodevelopmental disorders are increasing worldwide, but their causes are only partly identified^7,8^. Organic phosphorus herbicides, a candidate for environmental hazards, are widely used to control unwanted plants and increase crop yields. However, the developmental neurotoxic effects of these herbicides are still unclear. Particularly, glufosinate-ammonium (GLA), also known as 2-amino-4-[hydroxy(methyl)phosphoryl] butanoate, is a broad spectrum organophosphorus herbicide used worldwide^9^. GLA is an irreversible inhibitor of the glutamine synthetase required for plants to synthesize glutamine created by introducing ammonia into glutamate^10^. Due to the glutamine synthetase expression in astrocytes in the mammalian brain, GLA facilitates neurotoxicity including spatial memory impairments and epileptic activity^11,12^. GLA was developed for genetically modified crops resistant to herbicides and is the first alternative herbicide for glyphosate sprayed on herbicide-resistant crops^9^. As a result, GLA usage is increasing rapidly, but the risks of abnormal cortical development are still unclear. Therefore, this study investigates the effects of perinatal exposure to GLA on cortical development.

## Materials and methods

### Ethics statement

All experimental protocols were approved by the Animal Care and Use Committee of the Korea Institute of Toxicology (KIT) and comply with the Association for Assessment and Accreditation of Laboratory Animal Care International Animal Care Policies (Approval No. 1806-233, 1808-0924).

### Animal care and oral administration of glufosinate-ammonium

Wild-type Sprague Dawley rats were purchased from OrientBio (Sung-Nam, Korea). The temperature, humidity, and light cycle of animal room were automatically controlled by central control system (23–26 °C, relative humidity 50 ± 10%, 12 h light/12 h dark cycle with 150–300 lx). The solid diets (Lab Diet #5053, PMI Nutrition International, St. Louis, MO) were provided to the animals ad libitum. The day a vaginal plug was detected was designated gestation day 0 (GD 0) and the offspring’s birthday was designated postnatal day 0 (PND 0). GLA (Combi-Blocks, San Diego, CA) dissolved in normal saline (Dai Han Pharm., Seoul, Korea) and administered orally (0, 100, 250 mg/kg) to pregnant females from the time of implantation (GD 6) throughout lactation (PND 21). Offspring identification and culling were performed at PND 4.

### Righting reflex

Righting reflex tests were performed as described previously^13^. At PND 6, pups were placed on their backs on a bench pad and held in position for 5 s. The time required to return to the prone position was recorded. The righting reflex index was scored as follows: 0 points for success within 5 s (fast), 1 point for success in less than 15 s (moderate), and 2 points for success over 15 s (late). Righting reflex tests were repeated for 3 trials with 1 min intervals.

### Grip strength

Grip strength tests were performed as described previously^13^. At PND 15, pups were placed on wire mesh and held in position for 5 s. To test the pups’ ability to resist gravity, the wire mesh was inverted to 30, 60, 90, 120, and 150 degrees for 10-s intervals at each position. The time and degree at which the pups fell off the mesh were recorded. Grip strength indexes were scored as follows: 0 points for resisting less than 10 s, 1 point for resisting less than 20 s, 2 points for resisting less than 30 s, 3 points for resisting less than 40 s, 4 points for resisting less than 50 s, and 5 points for resisting over 50 s. A hanging impulse was calculated as the weight (g) × latency to fall (s), reflecting the force needed to resist gravity. Grip strength tests were repeated for 3 trials for each pup.

### The open field test

Open field tests were performed at PND 17 using a method previously described^14,15^. The open field arena consisted of 25 × 25 × 30 cm square acryl boxes. Rats were acclimated in the testing room for 30 min prior to starting the test. Rats were placed in the center of the open field arena and left to freely explore for 15 min for the test session with a video recording system. Test chambers were cleaned with 70% isopropanol and distilled water before each session. The average speed, total distance, mobility rate, and time spent on the edge were automatically analyzed using ToxTrac program^16^.

### Rotarod test

Accelerating rotarod tests were performed as described previously^17,18^. At postnatal week 7, rats were tested on a rotarod (Panlab Harvard Apparatus, Barcelona, Spain) accelerating from 4 to 40 rpm in 300 s. Rats were evaluated for 9 trials per session. At least 180 s of resting time was allowed between each trial. The end of a trial was determined when rats fell off the rod or when they reached 300 s. The latency to fall, speed, and time were recorded for each trial.

### Immunofluorescence staining

Immunofluorescence staining was performed as described previously^19^. For immunohistochemistry, GD 18 embryo brains were dissected and fixed with 4% paraformaldehyde (PFA) for 18 h. After fixation, brains were washed with phosphate buffered solution overnight and incubated with a 30% sucrose solution for 18 h for cryoprotection. The following antibodies were used: rabbit anti-Sox2 (Abcam, Cambridge, MA; 1:1000), mouse anti-Tuj1 (Millipore, Burlington, MA; 1:1000), and rabbit anti-Calbindin D (Swant, Switzerland; 1:2000). For immunocytochemistry, cells were fixed with 4% PFA, then stained with rabbit anti-Sox2 (Abcam, Cambridge, MA; 1:1000) and mouse anti-Tuj1 (Millipore, Burlington, MA; 1:1000). For F-actin staining, Alexa Fluor 488 conjugated phalloidin (Invitrogen, Carlsbad, CA; 1:1000) was used. Appropriate fluorophore-conjugated secondary antibodies (Invitrogen, Carlsbad, CA) were used with 4′,6-diamidino-2-phenylindole (DAPI) mounting medium (Abcam, Cambridge, MA) for nuclear staining. All images were acquired using an LSM-800 confocal microscope with ZEN software (Zeiss, Oberkochen, Germany).

### Primary cortical neuron culture

Primary cortical neuron cultures were developed as described previously^20^. GD 18 rat embryo cortices were isolated and dissected with trypsin. The cortical neurons were then plated on coverslips coated with 100 mg/mL poly d-Lycine (Sigma, St. Louis, MO) in neurobasal media supplemented with B27 (Invitrogen, Carlsbad, CA) and cultured for 1–7 days. For the neuronal viability test, Cell Counting Kit-8 (Dojindo Molecular Technologies, Rockville, MD) was used according to the manufacturer’s protocol; absorbance was measured at 450 nm/640 nm using a SynergyMx microplate reader (BioTek, Winooski, VT). Data are represented as the means of triplicate values; trials were repeated independently at least 3 times.

### Quantification and statistical analyses

Statistical analyses were performed using SigmaStat 3.5 software (Systat Software, San Jose, CA). Tests performed before e-weaning stage including righting reflex, grip strength, open field test were analyzed both male and female together using a Kruskal–Wallis one-way analysis of variance on ranks with the Dunn’s method. The rotarod test was conducted the male and female were analyzed separately using a Kruskal–Wallis one-way analysis of variance on ranks with the Dunn’s method. To count the Sox2^+^ and calbindin D^+^ cells, at least 3 embryos were analyzed for each group. The number of Sox2-expressing cells were automatically measured in a 500 × 500 pixel area of the ventricular zone using an ImageJ program with an Image-based Tool for Counting Nuclei plugin (National Institutes of Health, Bethesda, MD). The number of calbindin D-expressing cells were counted in a cortical plate 300 pixels wide. To measure neurite length, primary cortical neurons were harvested at 1 day in vitro (DIV) and Tuj1^+^ neurites were automatically analyzed using ZEN software (Zeiss, Oberkochen, Germany). Cortical neuron cultures were repeated independently at least 3 times. Statistical significance was considered when p < 0.05 for * or p < 0.001 for ***. Values are expressed as the mean ± the standard error of the mean.

## Results

### Perinatal exposure of glufosinate-ammonium reduced postnatal body weight but pups recovered by postnatal day 21

**Figure 1:**
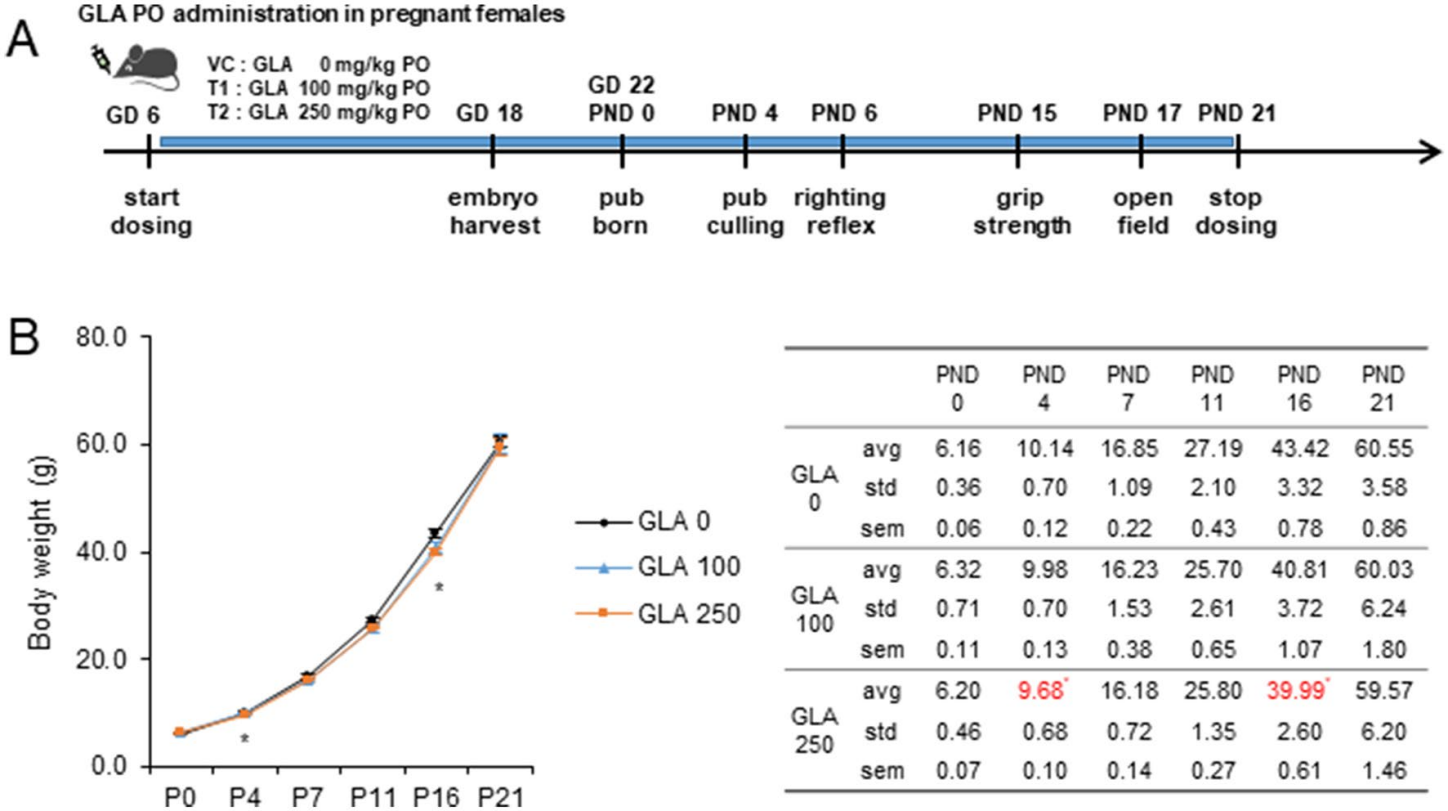
Experimental procedure of neurotoxicity tests for GLA. (**A**) Sprague Dawley rats were treated by oral gavage with 0, 100, or 250 mg/kg of GLA from gestation day (GD) 6 to postnatal day (PND) 21. (**B**) Weight changes of the offspring are shown. Offspring weights were reduced at PND 4 and PND 16. Error bars represent SD; n.s., not significant; ***p < 0.05 compared with controls, Kruskal–Wallis one-way analysis of variance on ranks with Dunn’s method (GLA 0 mg/kg, n = 16–23; GLA 100 mg/kg, n = 12–16; GLA 250 mg/kg, n = 18–24); *GLA* glufosinate-ammonium; *SD* standard deviation.

To investigate whether GLA exposure during perinatal periods induced brain development changes, pregnant Sprague Dawley rats were orally administered 0, 100, or 250 mg/kg GLA from GD 6 to PND 21 (Fig. 1A). Maternal body weight was measured from GD 6 to PND 21. There were no significant difference during pregnant stage and lactation stage (Supplementary Fig. S1A). The weight of the pups was monitored from PND 0 to 21. At PND 0, GLA-exposed pups did not differ in weight (GLA 0 mg/kg, 6.16 ± 0.06 g, GLA 100 mg/kg, 6.32 ± 0.11 g, GLA 250 mg/kg, 6.20 ± 0.07 g). However, at PND 4 and PND 16, pups exposed to 250 mg/kg GLA exhibited decreases in weight (PND 4: GLA 0 mg/kg, 10.14 ± 0.12 g; GLA 100 mg/kg, 9.98 ± 0.13 g; GLA 250 mg/kg, 9.68 ± 0.10 g; p < 0.05, PND 16: GLA 0 mg/kg, 43.42 ± 0.78 g; GLA 100 mg/kg, 40.81 ± 1.07 g; GLA 250 mg/kg, 39.99 ± 0.61 g; p < 0.05). GLA-exposed pups grew to be similar in weight to controls by PND 21 (GLA 0 mg/kg, 60.55 ± 0.86 g; GLA 100 mg/kg, 60.03 ± 1.80 g; GLA 250 mg/kg, 59.57 ± 1.46 g) (Fig. 1B).

### Perinatal exposure of glufosinate-ammonium induced an abnormal righting reflex response and motor coordination at postnatal day 6

**Figure 2:**
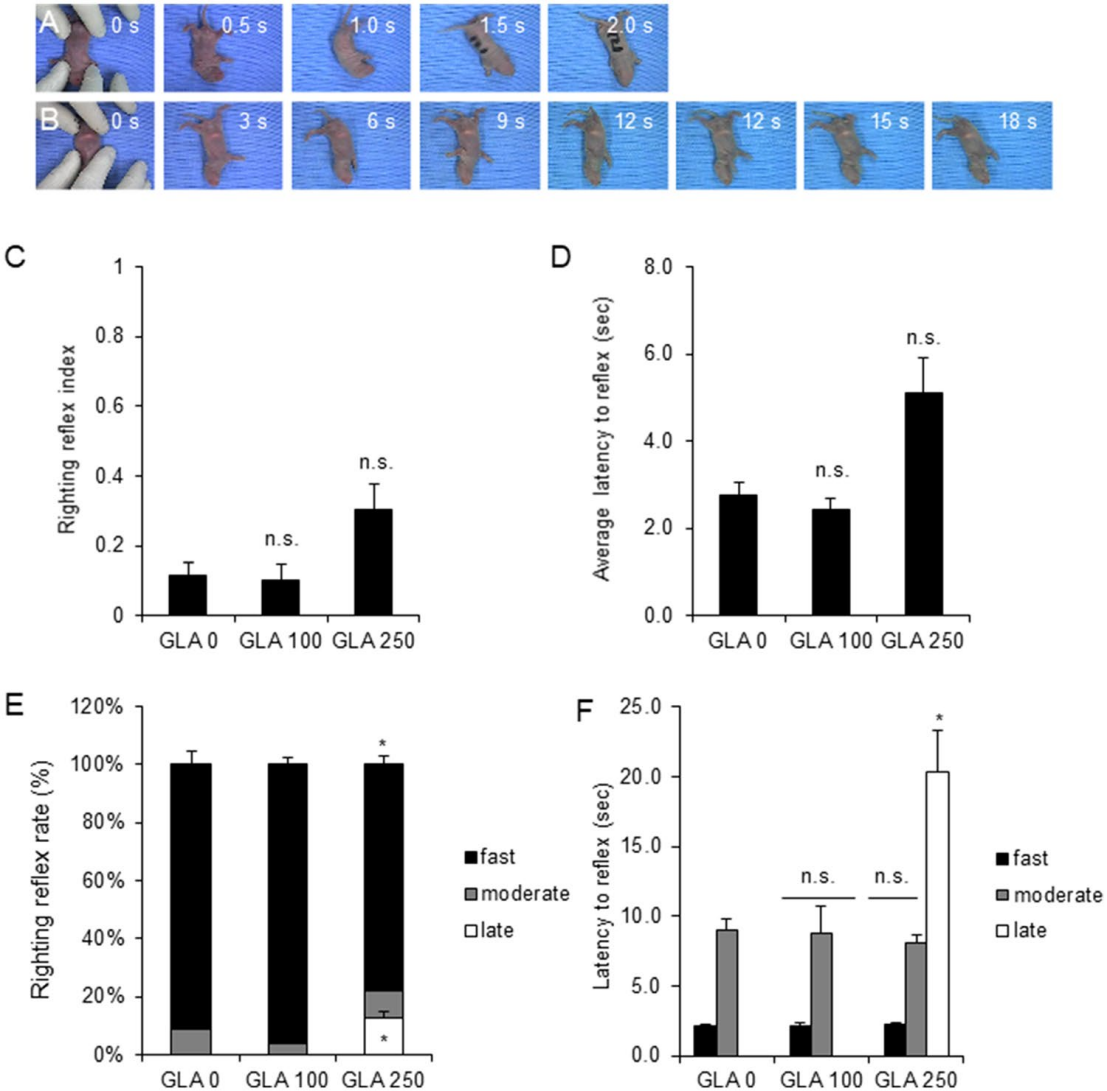
Effect of prenatal GLA exposure on the righting reflex in Sprague Dawley rats. (**A**,**B**) An example of the righting reflex test at PND 6 is shown. Pups were placed on their back on a surface; high-dose pups (**B**) showed longer recovery times compared with control pups (**A**). (**C**–**F**) Righting reflex test results are quantified. The latency to reflex in high-dose groups increased, especially above 15 s. Error bars represent the S.E.M.; n.s., not significant; *p < 0.05 compared with controls, Kruskal–Wallis one-way analysis of variance on ranks with Dunn’s method (GLA 0 mg/kg, n = 23; GLA 100 mg/kg, n = 16; GLA 250 mg/kg, n = 24); *GLA* glufosinate-ammonium; *PND* postnatal day; *S.E.M.* standard error of the mean.

Since decreases in weight can facilitate a developmental delay in motor functions, motor coordination was tested at PND 6 using a righting reflex test to investigate the ability of pups to flip onto their feet from the supine position (Fig. 2A,B). A righting reflex test can examine trunk control abilities and postural imbalances^13,21,22^. The righting reflex index value and the average latency to reflex were not different between controls and GLA-treated pups (righting reflex index: GLA 0 mg/kg, 2.77 ± 0.27 s, GLA 100 mg/kg, 2.44 ± 0.25 s; GLA 250 mg/kg, 4.59 ± 0.92 s; average latency to reflex: GLA 0 mg/kg, 2.77 ± 0.27 s; GLA 100 mg/kg, 2.44 ± 0.25 s; GLA 250 mg/kg, 4.59 ± 0.92 s) (Fig. 2C,D). Though their average latency was not altered, several pups exposed to 250 mg/kg GLA exhibited a long latency to flip from the supine position. These pups tried to return to the prone position constantly but failed to achieve the prone position rapidly.

To identify motor coordination defects, pups were divided into three groups according to flip latency (fast, moderate, and late); further analysis focused on the late latency group. Especially, late latency pups were not present in the GLA 0 mg/kg or GLA 100 mg/kg groups, but late latency group population increased and the latency to reflex also dramatically increased among GLA 250 mg/kg pups (late latency: 8.33 ± 2.41%, p < 0.05; latency of reflex: 23.47 ± 3.84 s, p < 0.05). (Fig. 2E,F). These data indicate that GLA exposure during the perinatal periods may lead to weakness in the limbs and trunk muscles as well as a motor coordination imbalance.

### Perinatal exposure to glufosinate-ammonium decreases the hanging impulse at postnatal day 15

**Figure 3:**
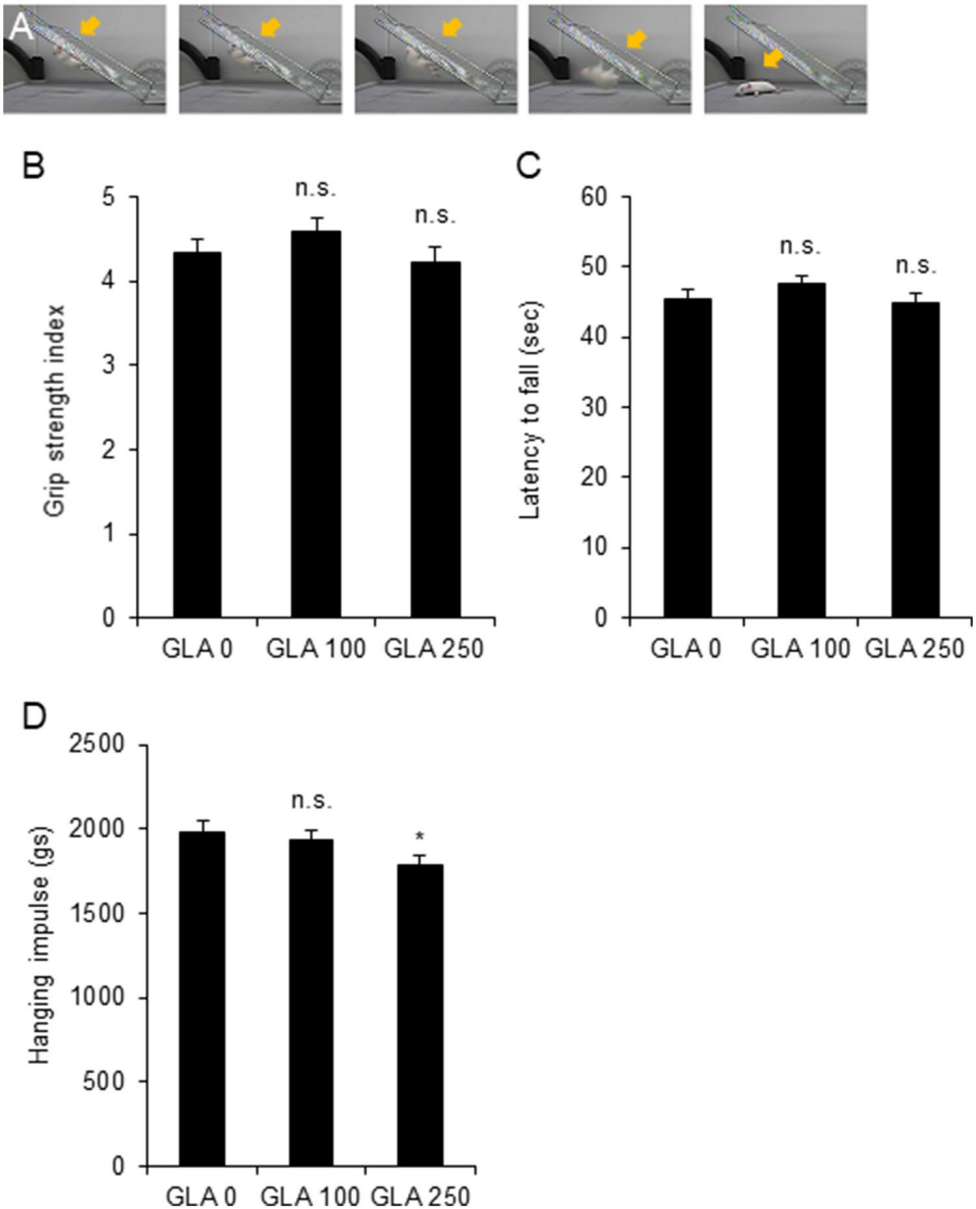
Effect of prenatal GLA exposure on the grip strength of Sprague Dawley rats. (**A**) An example of the grip strength test at PND 15. (**B**–**D**) Grip strength test results are quantified at PND 15. The hanging impulse in high-dose groups decreased. Error bars represent the S.E.M; n.s., not significant; *p < 0.05 compared with control, Kruskal–Wallis one-way analysis of variance on ranks with Dunn’s method (GLA 0 mg/kg, n = 18, s; GLA 100 mg/kg, n = 12; GLA 250 mg/kg, n = 18); *GLA* glufosinate-ammonium; *PND* postnatal day; *S.E.M.* standard error of the mean.

Due to the abnormal righting reflex response at PND 6, muscle weakness in all 4 limbs was evaluated by a grip strength test at PND 15 (Fig. 3A); this test assessed the ability of pups to resist falling from a wire mesh. The grip strength index and the latency to fall were similar between controls and GLA-exposed pups (grip strength index: GLA 0 mg/kg, 4.32 ± 0.16, GLA 100 mg/kg, 4.58 ± 0.17; GLA 250 mg/kg, 4.22 ± 0.18; latency to fall: GLA 0 mg/kg, 45.44 ± 1.28 s; GLA 100 mg/kg, 47.50 ± 1.13 s; GLA 250 mg/kg, 44.73 ± 1.37 s) (Fig. 3B,C). Also, the hanging impulse was calculated to reflect the force needed to resist gravity. As the hang time involves maintaining a minimum force required to oppose the gravitational force, a hanging impulse is an advantageous analytical tool for measuring phasic tension^22^. Pups exposed to 250 mg/kg GLA displayed a decreased hanging impulse (GLA 0 mg/kg, 1981.32 ± 63.76 gs; GLA 100 mg/kg, 1936.84 ± 55.78 gs; GLA 250 mg/kg, 1783.48 ± 54.58 gs; p < 0.05) (Fig. 3D) and indicated that there is a significant deficit in grip strength by perinatal GLA exposure.

### Perinatal exposure to glufosinate-ammonium leads to locomotor activity changes at postnatal day 17

**Figure 4:**
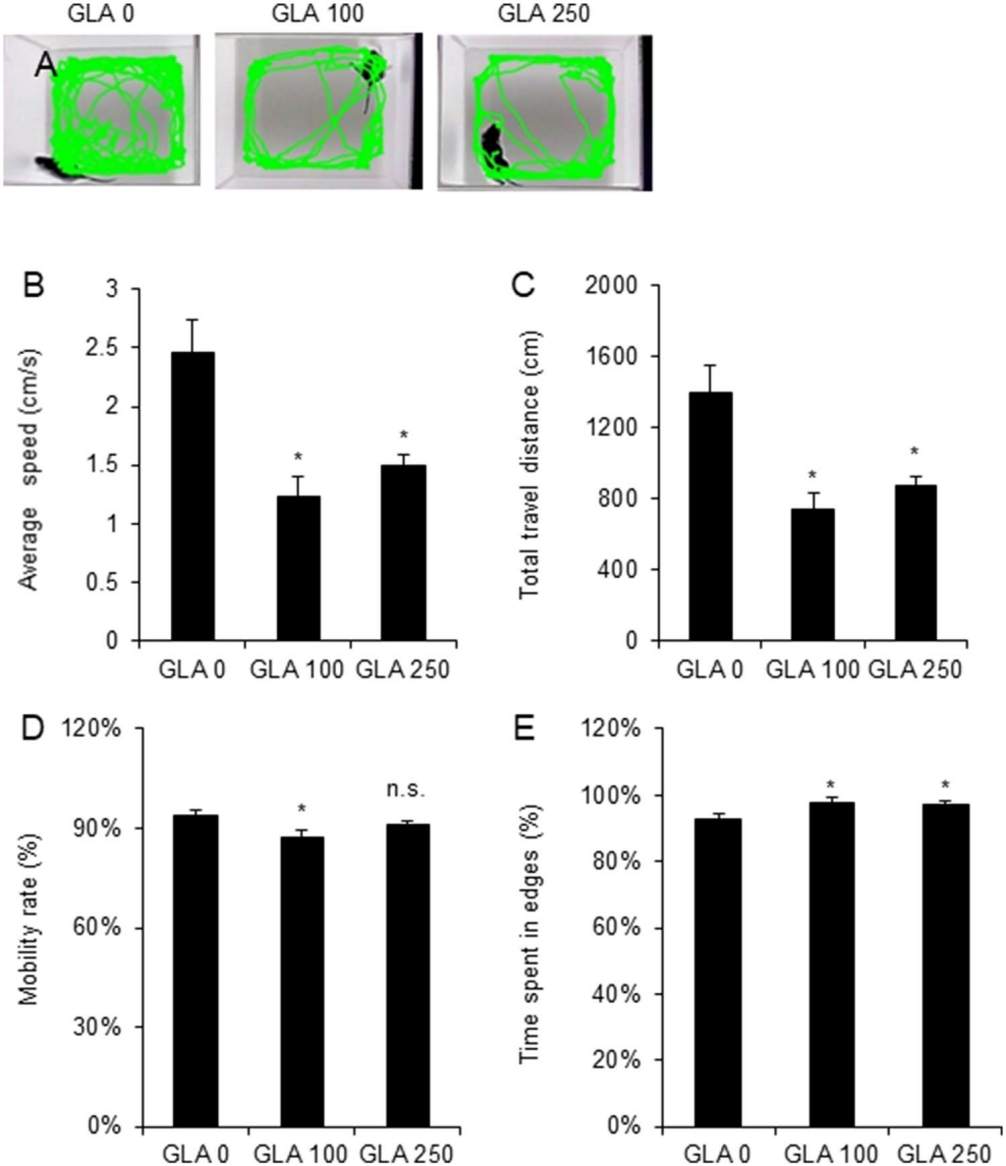
Effect of prenatal GLA exposure on the open field test in PND 16 Sprague Dawley rats. (**A**) An illustrative example of a pup’s travel trajectory in the open field test. (**B**–**E**) Quantification of open field test results at PND 16. GLA 100 mg/kg and 250 mg/kg groups showed decreased mobility. Error bars represent the S.E.M.; n.s., not significant; *p < 0.05 compared with controls, Kruskal–Wallis one-way analysis of variance on ranks with Dunn’s method (GLA 0 mg/kg, n = 18; GLA 100 mg/kg, n = 12; GLA 250 mg/kg, n = 18); *GLA* glufosinate-ammonium; *PND* postnatal day; *S.E.M.* standard error of the mean.

An open field test at PND17 was performed to identify whether perinatal GLA exposure also affects pup mobility. Rat pups freely explored open field boxes for 15 min in and their mobility was analyzed; GLA exposure during the perinatal stage affected pup mobility (Fig. 4A). Interestingly, 100 mg/kg of GLA exposure also decreased the average speed and total travel distance compared to 250 mg/kg of GLA exposure (average speed: GLA 0 mg/kg, 2.45 ± 0.29 cm/s; GLA 100 mg/kg, 1.24 ± 0.17 cm/s; GLA 250 mg/kg, 1.50 ± 0.09 cm/s; total travel distance: GLA 0 mg/kg, 1394.01 ± 155.2 cm; GLA 100 mg/kg, 742.37 ± 93.43 cm; GLA 250 mg/kg, 880.26 ± 49.05 cm; p < 0.05) (Fig. 4B,C). Also, the mobility rate was only reduced in 100 mg/kg GLA-exposed pups (GLA 0 mg/kg, 93.74 ± 1.67%; GLA 100 mg/kg, 87.21 ± 2.19%, p < 0.05; GLA 250 mg/kg, 91.06 ± 1.29%) (Fig. 4D). An analysis of the time spent on the edges during the open field area exploration revealed that all GLA-exposed pups spent significantly more time on the edges of the maze compared to control pups (GLA 0 mg/kg, 92.75 ± 1.43%; GLA 100 mg/kg, 97.87 ± 1.40%; GLA 250 mg/kg, 97.43 ± 0.63%; p < 0.05) (Fig. 4E).

### Perinatal exposure to glufosinate-ammonium induced abnormal motor coordination and balance in male rats

**Figure 5:**
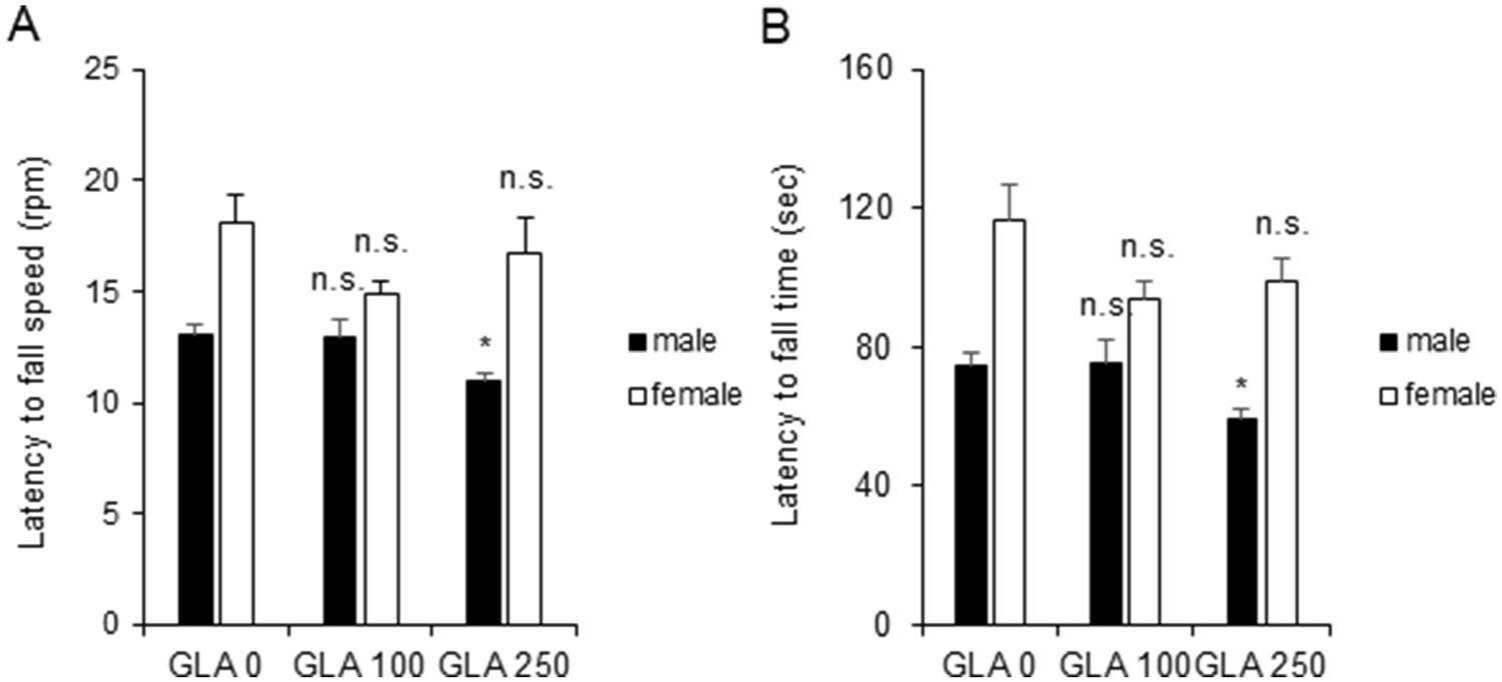
Effect of perinatal GLA exposure on the rotarod performance test in juvenile Sprague Dawley rats. (**A**,**B**) Quantification of rotarod test results at PNW 7. GLA 250 mg/kg groups of male pups showed a smaller latency. Kruskal–Wallis one-way analysis of variance on ranks with Dunn’s method; (GLA 0 mg/kg, n = 6; GLA 100 mg/kg, n = 4; GLA 250 mg/kg, n = 6 each sex); *GLA* glufosinate-ammonium; *PNW* postnatal week; *S.E.M.* standard error of the mean.

Accelerating rotarod tests were conducted at postnatal week 7 to examine whether perinatal GLA exposure causes motor coordination and balance impairments; the latency to fall speed and time were measured. Male pups exposed to 250 mg/kg GLA showed significantly abnormal motor coordination and balance (latency to fall speed: GLA 0 mg/kg, 13.02 ± 0.48 rpm; GLA 100 mg/kg, 12.97 ± 0.81 rpm; GLA 250 mg/kg, 10.98 ± 0.35 rpm; latency to fall time: GLA 0 mg/kg, 74.98 ± 3.60 s; GLA 100 mg/kg, 75.35 ± 6.12 s; GLA 250 mg/kg, 59.51 ± 2.82 s; p < 0.05) (Fig. 5A,B). These data indicate early motor coordination defects persist until the juvenile stage in male rats after perinatal GLA exposure.

### Abnormal cortical interneuron development after perinatal exposure to glufosinate-ammonium

**Figure 6:**
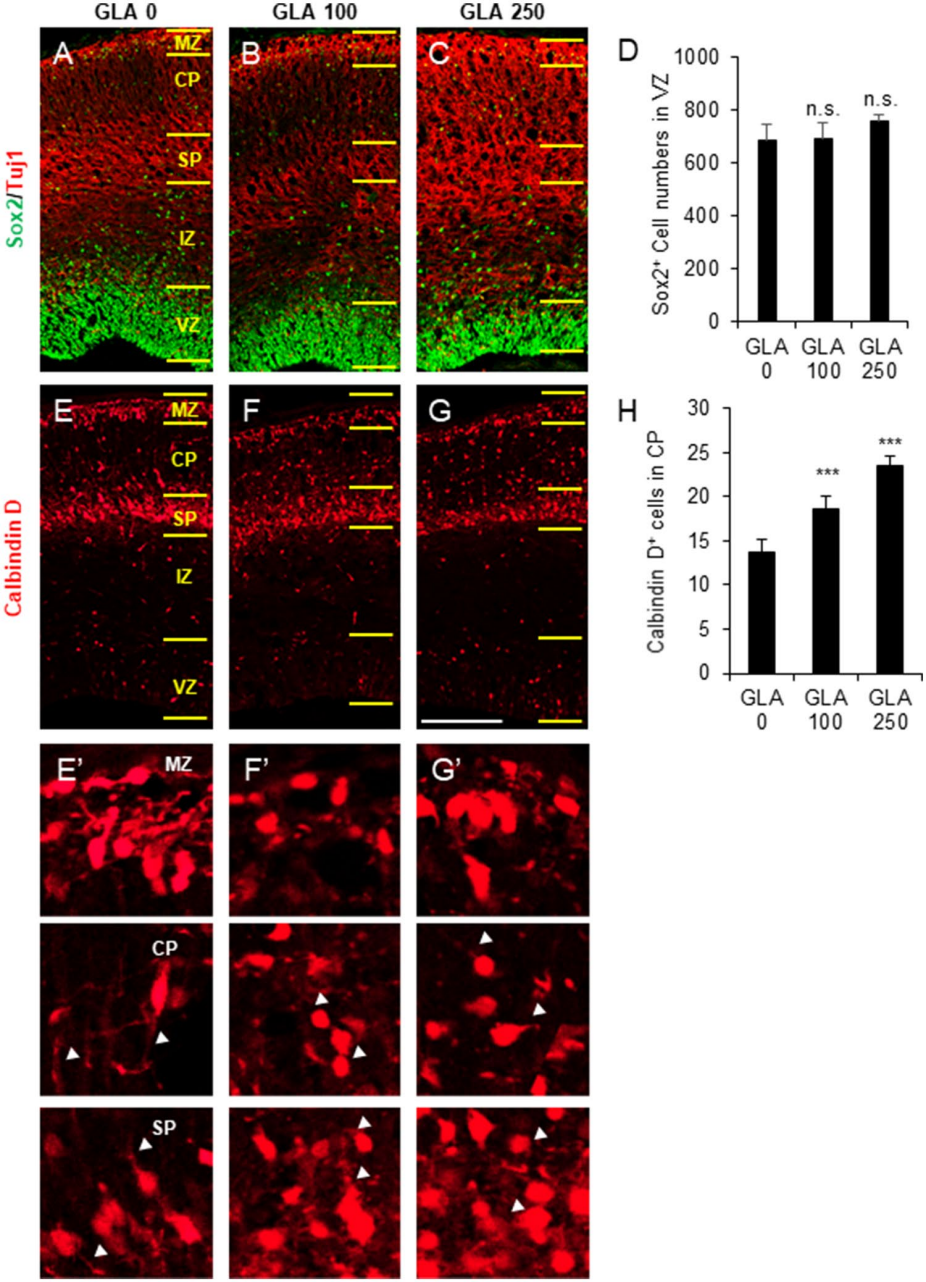
Abnormal cortical interneuron migration after perinatal exposure to GLA. (**A**–**C**) Immunohistochemistry of Sox2^+^ neural progenitors and Tuj1^+^ neurons on sections of the telencephalon. (**D**) Quantification of Sox2^+^ cells in the ventricular zone (> 3 embryos in each group). (**E**–**G**) Immunohistochemistry of calbindin D^+^ in telencephalon interneurons and their magnification views (**E’**–**G’**). Calbindin D^+^ neurons and layer 2/3 neuron positions were changed after perinatal GLA exposure. Arrowhead indicated axon of Calbindin D^+^ neurons. (H) Calbindin D^+^ cell quantification in the cortical plate (> 3 embryos in each group) is shown. Error bars represent the S.E.M.; n.s., not significant; *p < 0.05, ***p < 0.001 compared with controls, Kruskal–Wallis one-way analysis of variance on ranks with Dunn’s method. Scale bars: G, 100 μm. *MZ* marginal zone; *CP* cortical plate; *SP* subplate; *IZ* intermediate zone; *VZ* ventricular zone; *GLA* glufosinate-ammonium; *PND* postnatal day; *S.E.M.* standard error of the mean.

To address the underlying mechanism of motor coordination defects, rat premotor cortical areas were analyzed at GD 18. First, cortical progenitor cells in the cortex were investigated regarding whether neural progenitor cell survival and proliferation were altered by perinatal GLA exposure. Immunohistochemistry was performed with a Sox2 antibody for neural progenitor cells and a Tuj1 antibody for cortical neurons (Fig. 6A–C). In the ventricular zone, the number of Sox2^+^ neural progenitor cells did not change after GLA exposure (GLA 0 mg/kg, 685.75 ± 57.65 cells; GLA 100 mg/kg, 693.50 ± 57.02 cells; GLA 250 mg/kg, 755.25 ± 26.87 cells) (Fig. 6D). These data indicate that GLA exposure did not affect neural progenitor cell survival or proliferation.

Abnormal interneuron development, including migration and circuit formation, clearly affect motor function^23,24^. To identify whether cortical interneurons are intact after GLA exposure, interneuron development in the motor cortical area was examined. Immunohistochemistry was performed with a calbindin D antibody for cortical interneurons (Fig. 6E–G). Most calbindin D^+^ interneurons were differentiated from the ganglionic eminence and migrated to the marginal zone and subplate at embryonic day 16–18 in the developing mouse cortex. After GLA exposure, more calbindin D^+^ interneurons were present in the cortical plate (GLA 0 mg/kg, 13.75 ± 1.46 cells; GLA 100 mg/kg, 18.63 ± 1.41 cells; GLA 250 mg/kg, 23.50 ± 1.09 cells) (Fig. 6H). Especially, calbindin D^+^ interneurons were reduced in the marginal zone and subplate; the total number of interneurons did not change. These numbers indicate that interneuron migration from the ganglionic eminence was disrupted by GLA exposure. In addition, the axon lengths of calbindin D^+^ interneurons were slightly reduced after GLA exposure. (Fig. 6E’-G’ arrow heads) Thus, perinatal GLA exposure disrupted cortical interneuron migration and axon outgrowth.

### Glufosinate-ammonium disrupts the neurite outgrowth of cortical neurons in vitro

**Figure 7:**
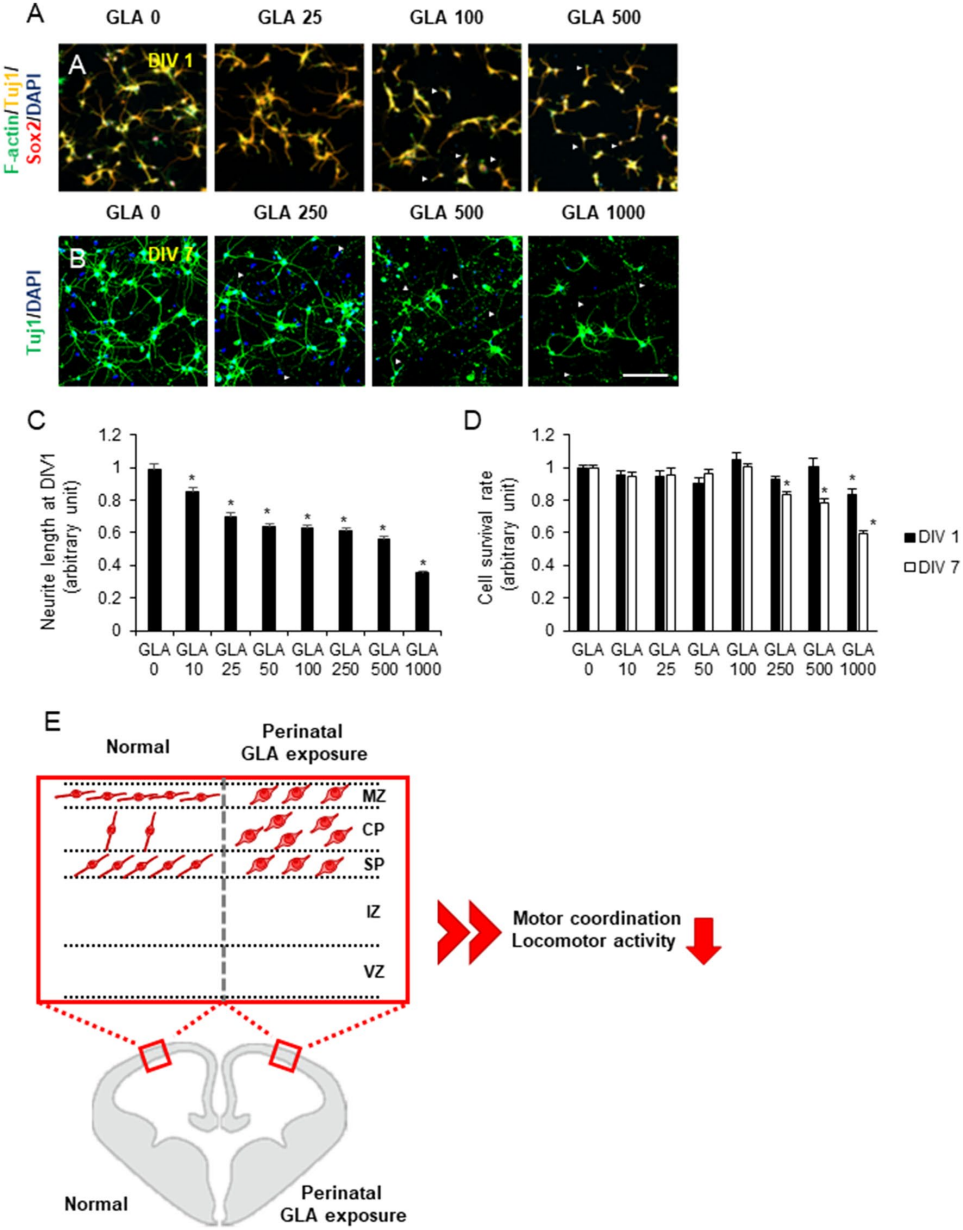
Effect of GLA on neurite outgrowth. (**A**,**B**) Immunocytochemistry of primary cortical neurons at 1 and 7 DIV. Various GLA concentrations were added to the culture medium. Arrowhead indicated axonal degeneration. (**C**) Tuj1^+^ neurite length quantification at 1 DIV is shown. GLA reduced neurite outgrowth in a concentration-dependent manner. (**D**) Neuronal viability quantification at 1 and 7 DIV is shown. (**E**) A schematic diagram of abnormal cortical development of perinatal GLA exposure is depicted. Error bars represent the S.E.M; n.s., not significant; *p < 0.05 compared with controls; Kruskal–Wallis one-way analysis of variance on ranks with Dunn’s method. Scale bars: **B**, 100 μm; *GLA* glufosinate-ammonium; *DIV* days in vitro; *S.E.M.* standard error of the mean.

Given that perinatal GLA exposure disrupted cortical interneurons migration and axon outgrowth, possible reductions in axon outgrowth by GLA treatment were evaluated. Primary cortical neurons were plated with various GLA concentrations and neurite outgrowth was examined after 24 h by Tuj1 immunostaining (Fig. 7A). When grown in vitro in the presence of GLA, neurite lengths were reduced by a GLA concentration-dependent manner (GLA 0 mg/mL, 1.00 ± 0.03 fold; GLA 10 mg/mL, 0.85 ± 0.03 fold; GLA 25 mg/mL, 0.70 ± 0.02 fold; GLA 50 mg/mL, 0.64 ± 0.02 fold; GLA 100 mg/mL, 0.63 ± 0.02 fold; GLA 250 mg/mL, 0.61 ± 0.01 fold; GLA 500 mg/mL, 0.56 ± 0.01 fold; GLA 1000 mg/mL, 0.36 ± 0.01 fold) (Fig. 7A,C). More specifically, in the presence of 250 mg/mL GLA, axon and dendrite degeneration were observed at 7 DIV (Fig. 7B).

To address whether GLA affected cortical neuron survival, neuronal viability was examined at 1 and 7 DIV. At 1 DIV, neuronal viability was only reduced in the presence of 1000 mg/mL GLA (GLA 0 mg/mL, 1.00 ± 0.01 fold; GLA 10 mg/mL, 0.96 ± 0.02 fold; GLA 25 mg/mL, 0.95 ± 0.04 fold; GLA 50 mg/mL, 0.91 ± 0.03 fold; GLA 100 mg/mL, 1.05 ± 0.05 fold; GLA 250 mg/mL, 0.93 ± 0.02 fold; GLA 500 mg/mL, 1.01 ± 0.05 fold; GLA 1000 mg/mL, 0.84 ± 0.03 fold). However, at 7 DIV, neuronal viability was reduced at GLA values above 250 mg/mL (GLA 0 mg/mL, 1.00 ± 0.01 fold; GLA 10 mg/mL, 0.95 ± 0.03 fold; GLA 25 mg/mL, 0.96 ± 0.04 fold; GLA 50 mg/mL, 0.97 ± 0.02 fold; GLA 100 mg/mL, 1.00 ± 0.02 fold; GLA 250 mg/mL, 0.83 ± 0.02 fold; GLA 500 mg/mL, 0.78 ± 0.03 fold; GLA 1000 mg/mL, 0.60 ± 0.01 fold) (Fig. 7D). In summary, the presence of GLA disrupted neurite outgrowth and ultimately affected neuronal viability. Taken together, these results provide new evidence that early life exposure to the GLA might affect the cortical development and facilitate behavioral changes (Fig. 7E).

## Discussion

In this study, perinatal GLA exposure induced abnormal motor coordination by disrupting cortical interneuron migration. Cortical circuits consist of glutamatergic excitatory neurons and γ-aminobutyric acid (GABA)ergic inhibitory interneurons. The balance of excitatory and inhibitory neurons is important for proper function; changes in this neural balance could cause multiple neurodevelopmental disorders in humans^3–6^. Meanwhile, evidence suggests that GABAergic inhibitory interneurons in the primary motor cortex directly control voluntary movement^25–29^. In this study, perinatal GLA exposure induced weakness of the limbs and trunk muscles as well as a motor coordination imbalance in rats. Decreased body weight in pups raises the possibility of delayed development, but the adolescent motility impairment demonstrated by the rotarod test supported the possibility that GLA directly affected the balance of excitatory and inhibitory during brain development. Also, perinatal GLA exposure decreased motility and increased the time spent on the edges in the open field test. Since altered GABAergic inhibitory interneuron development could affect developmental disorders including autism and intellectual disabilities, a relationship with these diseases should be investigated.

Moreover, perinatal GLA exposure disrupted the migration of interneurons expressing calbindin D. Interestingly, abnormal migration of calbindin D^+^ interneurons showed a dose–response relationship with GLA administration. However, abnormal righting reflex response and decreases the hanging impulse only showed in the high dose group, locomotor activity decreased in the both groups. These data suggest that perinatal expose of GLA may have influenced the other inhibitory neurons. Mature inhibitory interneuron subtypes can be divided by their markers, including parvalbumin, somatostatin, and serotonin receptor 3A^30^. As immature calbindin D-expressing interneurons did not express their subtype marker at GD 18, interneuron subtypes were not distinguished. Further investigation should be performed to determine the type of inhibitory neurons that experience migration changes and the functional disability outcomes due to interneuron alterations after perinatal GLA exposure.

Mammalian glutamine synthetase regulates toxic ammonia and glutamate levels by converting glutamine^31,32^. Given that GLA acts a glutamine synthetase inhibitor, exposure to GLA could increase blood ammonia levels; also, hyperammonemia during pregnancy could lead to encephalopathy. However, perinatal GLA exposure did not induce a blood ammonia concentration in the dams in this study (Supplementary Fig. S1). This indicates GLA would not cause a change in the ammonia concentration in the fetal brain; thus, abnormal motor coordination and disrupted interneuron migration would not be due to GLA-related hyperammonemia. Meanwhile, it is well known that glutamine synthetase is expressed in the brain, especially astrocytes, but its expression in the developing cortex is still unclear^33^. We found that glutamine synthetase was expressed in developing cortical neurons (Supplementary Fig. S2). Notably, glutamate is a neurotransmitter in the brain that regulates the growth rate and branches of axons and dendrites during brain development^34,35^. This axon and dendrite outgrowth is an important process to establish functional neural circuits and neural migration^36^. Furthermore, glutamine synthetase inhibition could increase brain glutamate levels and lead to abnormal migration and neurite outgrowth in inhibitory neurons. It is not clear whether prenatal GLA exposure increases brain glutamate levels; hence, brain glutamate levels and the role of glutamine synthetase should be further investigated. In conclusion, the present results provide new evidence that early life exposure to the GLA might affect the cortical development and facilitate behavioral changes.

## Supplementary information

Supplementary Figure 1.

Supplementary Figure 1.

Supplementary Figure Legends.
