# Supplementary figures and images for: Impairment of motor coordination and interneuron migration in perinatal exposure to glufosinate-ammonium
Source: Sci Rep. 2020 Nov 26;10:20647. doi: 10.1038/s41598-020-76869-7 (PMC7691990; doi:10.1038/s41598-020-76869-7)

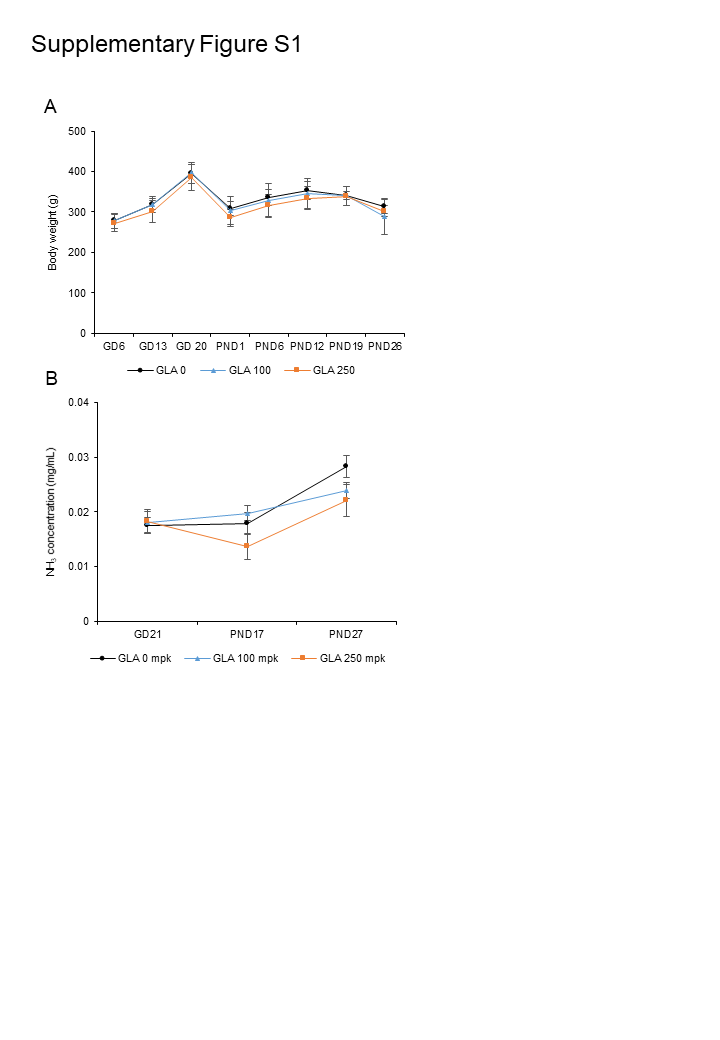

Supplement: Supplementary file 1 — Supplementary Figure 1. [file 41598_2020_76869_MOESM1_ESM.tif]

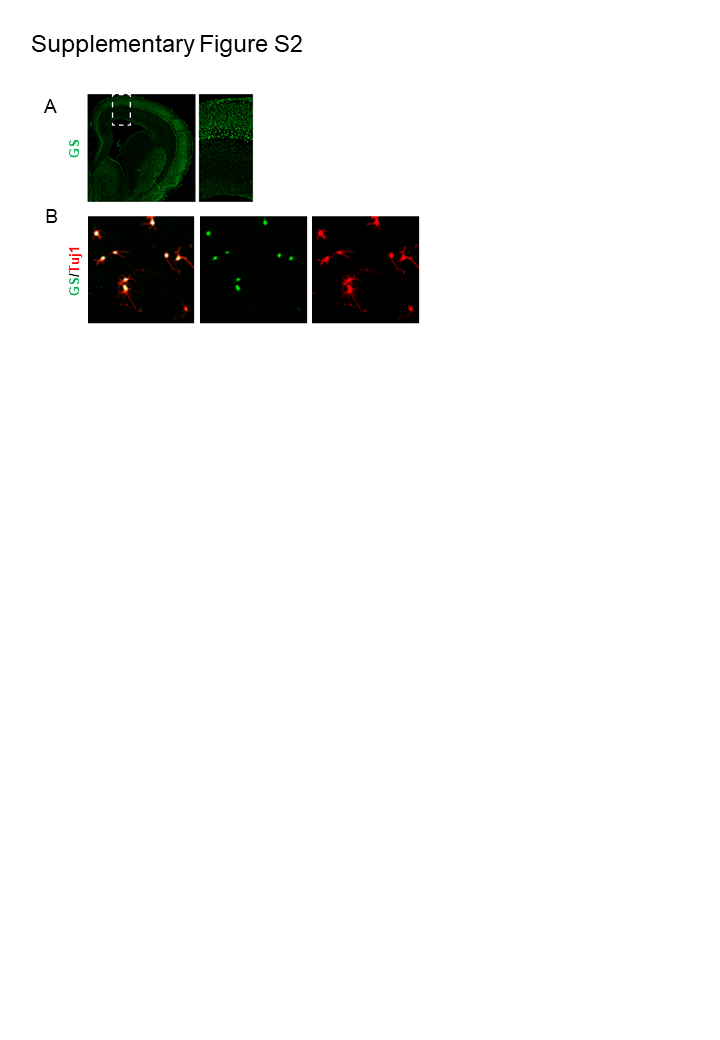

Supplement: Supplementary file 2 — Supplementary Figure 1. [file 41598_2020_76869_MOESM2_ESM.tif]
