# Supplementary material for: Impairment of motor coordination and interneuron migration in perinatal exposure to glufosinate-ammonium
Source: Sci Rep. 2020 Nov 26;10:20647. doi: 10.1038/s41598-020-76869-7 (PMC7691990; doi:10.1038/s41598-020-76869-7)
Supplement: Supplementary file 3 — Supplementary Figure Legends. [file 41598_2020_76869_MOESM3_ESM.docx]

**Supplementary information**

**Impairment of motor coordination and interneuron migration in perinatal exposure to glufosinate-ammonium**

**Kyung-Tai Kim^1^, Ye-Jung Kwak ^1^, Suchel Han^1*^, Jeong Ho Hwang^1*^**

**^1^** Jeonbuk Department of Inhalation Research, Korea Institute of Toxicology, 30 Baekhak1-gil, Jeongeup, Jeollabuk-do 56212, Republic of Korea

**Running title:** Glufosinate-ammonium affects motor coordination

**Keywords:** glufosinate-ammonium, motor coordination defects, cortical development, interneurons migration

|  |
| --- |

*Corresponding author

Suchel Han

Email: vethansc@kitox.re.kr

Phone: +82-63-570-8520

Fax: +82-63-570-8598

Jeong Ho Hwang

Email: jeongho.hwang@kitox.re.kr

Phone: +82-63-570-8528

Fax: +82-63-570-8598

**Supplementary Figure Legends**

**Supplementary Figure S1. Weight and blood ammonia concentration in dams.** (A) Weight change of dams from gestation day (GD) 6 to postnatal day (PND) 26. (B) Blood ammonia concentration of dams from GD21 to PND 27. There is not a statistically significant difference between the all groups. Error bars represent S.D., compared with control, Kruskal-Wallis one-way analysis of variance on ranks with Dunn’s method.

**Supplementary Figure S2. Immunostaining of glutamine synthetase in the developing cortex.** (A) Immunohistochemistry of glutamine synthetase in the rat cortex at GD 18 (B) Immunocytochemistry of glutamine synthetase in the primary cortical neurons at DIV1. Scale bars: 400 μm in A; 100 μm in B.

**Supplementary Materials and Methods**

**Immunofluorescence staining**

Immunofluorescence staining was performed as described previously^1,2^. The following antibodies were used: rabbit anti-glutamine synthetase (Abcam, Cambridge, MA; 1:1000), mouse anti-Tuj1 (Millipore, Burlington, MA; 1:1000). Appropriate fluorophore-conjugated secondary antibodies (Invitrogen, Carlsbad, CA) were used. All images were acquired using LSM-800 confocal microscope with ZEN software (Zeiss, Oberkochen, Germany).

**Blood ammonia concentration**

Dam's whole bloods were collected into heparin tubes at gastrulation day 21, postnatal day 17, and 27. After centrifugation, collected plasma was used for analysis of ammonia concentration. For measuring blood ammonia concentration, Ammonia Assay Kit (Abcam, Cambridge, MA) used by manufacture protocol, and the maximal absorbance was measured at 570 nm using a SynergyMx microplate reader (BioTek, Winooski, VT). Data represent as means of triplicate value.

**Quantification and statistical analyses**

Statistical analyses were performed using SigmaStat 3.5 software (Systat Software, San Jose, CA) and data were analyzed using a Kruskal-Wallis one-way analysis of variance on ranks with Dunn’s method. Values are expressed as mean ± standard deviation (SD).

**References**

1 Kim, K. T. *et al.* ISL1-based LIM complexes control Slit2 transcription in developing cranial motor neurons. *Sci Rep* **6**, 36491, doi:10.1038/srep36491 (2016).

2 Kim, K. T. & Song, M. R. Light-induced Notch activity controls neurogenic and gliogenic potential of neural progenitors. *Biochem Biophys Res Commun* **479**, 820-826, doi:10.1016/j.bbrc.2016.09.124 (2016).
